# Temporal changes in the levels of virus and betasatellite DNA in *B. tabaci* feeding on CLCuD affected cotton during the growing season

**DOI:** 10.3389/fmicb.2024.1410568

**Published:** 2024-05-22

**Authors:** Zafar Iqbal, Mariyam Masood, Muhammad Shafiq, Rob W. Briddon

**Affiliations:** ^1^Central Laboratories, King Faisal University, Al-Ahsa, Saudi Arabia; ^2^Department of Zoology, Government College Women University, Faisalabad, Pakistan; ^3^Department of Biotechnology, University of Management and Technology, Sialkot Campus, Sialkot, Pakistan; ^4^Agricultural Biotechnology Division, National Institute for Biotechnology and Genetic Engineering, Faisalabad, Pakistan

**Keywords:** cotton leaf curl disease, begomovirus, betasatellite, qPCR, titre, whitefly

## Abstract

Cotton, a key source of income for Pakistan, has suffered significantly by cotton leaf curl disease (CLCuD) since 1990. This disease is caused by a complex of phylogenetically-related begomovirus (genus *Begomovirus*, family *Geminiviridae*) species and a specific betasatellite (genus *Betasatellite*, family *Tolecusatellitidae*), *cotton leaf curl Multan betasatellite*. Additionally, another DNA satellite called alphasatellite (family *Alphasatellitidae*), is also frequently associated. All these virus components are vectored by a single species of whitefly (*Bemisia tabaci*). While many factors affect cotton productivity, including cotton variety, sowing time, and environmental cues such as temperature, humidity, and rainfall, CLCuD is a major biotic constraint. Although the understanding of begomoviruses transmission by whiteflies has advanced significantly over the past three decades, however, the in-field seasonal dynamics of the viruses in the insect vector remained an enigma. This study aimed to assess the levels of virus and betasatellite in whiteflies collected from cotton plants throughout the cotton growing season from 2014 to 2016. Notably, begomovirus levels showed no consistent pattern, with minimal variations, ranging from 0.0017 to 0.0074 ng.μg^–1^ of the genomic DNA in 2014, 0.0356 to 0.113 ng.μg^–1^ of the genomic DNA in 2015, and 0.0517 to 0.0791 ng.μg^–1^ of the genomic DNA in 2016. However, betasatellite levels exhibited a distinct pattern. During 2014 and 2015, it steadily increased throughout the sampling period (May to September). While 2016 showed a similar trend from the start of sampling (July) to September but a decline in October (end of sampling). Such a study has not been conducted previously, and could potentially provide valuable insights about the epidemiology of the virus complex causing CLCuD and possible means of controlling losses due to it.

## 1 Introduction

In Pakistan, cotton is the foremost cash crop and contributes up to 60% of foreign exchange earnings. Cotton is grown on approximately three times (2.5 million hectares) in Punjab province then the cotton grown area in Sindh province. Since the early 1990s the yield of cotton from the Punjab has been seriously reduced due to cotton leaf curl disease (CLCuD; Briddon and Markham, 2000). First reported near Multan, Punjab province in 1967 (Hussain and Ali, 1975), CLCuD remained localized until 1986 (Hussain and Mahmood, 1988; Hussain et al., 1991). It then ravaged cotton crops across Pakistan and northwestern India, causing a loss of US$5 billion to Pakistan’s economy (Briddon and Markham, 2000). By the late 1990s, resistant cotton varieties were introduced and gained some traction (Rahman et al., 2005), but succumbed to a new resistance-breaking strain in 2001. Subsequently, the resistance breaking “strain” of the disease spread into northwestern India (Mansoor et al., 2003a; Zaffalon et al., 2012; Sattar et al., 2013).

Cotton leaf curl disease (CLCuD) is caused by begomoviruses in association with a specific betasatellite known as cotton leaf curl Multan betasatellite (genus *Betasatellite*, family *Tolecusatellitidae*) (Briddon et al., 2001; Sattar et al., 2013). A number of distinct begomovirus species were shown to be associated with the disease in the 1990s, the most important of which are cotton leaf curl Multan virus (CLCuMuV) and cotton leaf curl Kokhran virus (CLCuKoV; Zhou et al., 1998; Mansoor et al., 2003b). These viruses are poorly infectious to *Gossypium hirsutum* and require CLCuMuB to cause typical CLCuD symptoms. After resistance breaking in 2001 the disease across the Punjab (Pakistan) in resistant cotton was shown to be associated with only a single virus, the “Burewala” strain of CLCuKoV (CLCuKoV-Bur); a strain resulting from recombination between CLCuKoV and CLCuMuV (Amrao et al., 2010a). Although this strain became dominant across the Punjab in Pakistan, in Sindh province Pakistan and northwestern India, although CLCuKoV-Bur was important, other virus species, some of which were not identified in the Punjab (Pakistan), persisted; likely due to the continued cultivation of non-resistant cotton varieties in these regions (Amrao et al., 2010b; Rajagopalan et al., 2012; Zaffalon et al., 2012). “Burewala” strain which consists of CLCuKoV-Bur in association with a recombinant CLCuMuB, known as the “Burewala” strain (CLCuMuB-Bur) (Amin et al., 2006). Notably, from 2015 to onward, researchers observed a significant change in the situation. There was a shift from the resistant strain to the resurgence of earlier CLCuD-associated begomoviruses in Pakistan (Zubair et al., 2017) and India (Datta et al., 2017). This shift foreshadowed a third CLCuD epidemic (Sattar et al., 2017; Mahmood et al., 2022).

Losses in agricultural productivity are greatly affected by a number of variables that include crop variety, sowing time, and environmental factors such as temperature, relative humidity, and rainfall. Biotic stresses, such as virus infection, further exacerbate these losses (Rejeb et al., 2014). For the virus complex causing CLCuD, and other diseases caused by geminiviruses, studies have addressed the effects of plant variety, sowing time and environmental factors such as temperature, relative humidity, and rainfall on disease incidence and crop loses (Tahir et al., 2004; Hussain et al., 2015). Similarly, studies have addressed the effects of environmental variables on whitefly populations and consequent effects on disease incidence and crop (yield) loses (Sharma et al., 2006; Hussain et al., 2015; Zeshan et al., 2015). However, although our understanding of the mechanism of transmission of, in this case, begomoviruses by the vector *Bemisia tabaci* has advanced significantly in the last 30 years (Czosnek et al., 2017). A significant knowledge gap remains regarding the seasonal dynamics of these viruses within their insect hosts in field conditions.

Several PCR-based methods, such as conventional PCR and multiplex PCR, along with sequence-based serological methods, such as enzyme linked immuno sorbent assay (ELISA), and hybridization-based methods, including dot blot and Southern blot, have become vital tool for the detection of begomoviruses (Boonham et al., 2004; Makkouk and Kumari, 2006; Accotto and Noris, 2007; Kushwaha et al., 2010; Visser et al., 2016). Nonetheless, of these methods, only quantitative real-time PCR (qPCR) provides the accuracy and sensitivity to detect the minute quantities of viral DNA harbored by insects (Boonham et al., 2002; Fabre et al., 2003; Olmos et al., 2005; Noris and Miozzi, 2015). This exceptional and unparalleled attributes of qPCR made it a prime choice for quantification of viral load.

The study described here was designed to investigate the levels of virus and betasatellite associated with CLCuD harbored by *B. tabaci* collected from cotton plants throughout the cotton growing season using qPCR. Such a study has not been conducted previously and could potentially provide valuable information about the epidemiology of the virus complex causing CLCuD and possible means of controlling losses due to it.

## 2 Materials and methods

### 2.1 Sample collection and DNA extraction

Whiteflies were collected from the vicinity of National Institute of Biotechnology and Genetic Engineering (NIBGE; 31° 39′ 6065″ N & 73° 02′ 7886″ E) in the cotton growing season from the year 2014 to 2016. Usually, cotton is sown in late April and remains in the field until the end of September. Samples were collected every month in the year 2014 and 2015 from May to September. In the year 2016 cotton was sown a month later than usual, so sampling was started in July and continued until October. Throughout the cotton growing season, whitefly samples were collected at fortnight intervals from symptomatic cotton plants. Two collections, each containing ten whiteflies, were taken on 1st and 15th days weeks of each month. The whiteflies samples were kept in 80% ethanol until use for DNA extraction. DNA was extracted using a Fast tissue to PCR kit (Thermo Fisher Scientific, USA) and DNA was quantified using NanoDrop spectrophotometer (Thermo Scientific Nanodrop Spectrophotometer 2000c). A working dilution (20 ng.μL^–1^) was made of each sample for qPCR. Non-viruliferous whiteflies, maintained in the insect rearing facility of NIBGE, were used as negative controls.

### 2.2 PCR amplification

The virus and betasatellite primers were designed in the conserved genome regions to amplify all the begomoviruses associated with CLCuD, including CLCuMuV, CLCuKoV-Bur, and CLCuMuB (Shafiq et al., 2017). To ensure efficient and reliable quantification by qPCR, PCR conditions, primers concentration and performance were first optimized using conventional PCR with DNA extracted from whiteflies.

### 2.3 Quantitative real-time PCR

The qPCR was conducted essentially as described in Shafiq et al. (2017). The reaction mixture for qPCR consisted of 12.5 μL SYBER Green Super mix, 0.25 μL (2.5 pico moles) of each primer, 2.5 μL of template DNA (50 ng.μL^–1^) and 9.5 μL of water to make a final volume 25 μL. The PCR cycling profile used was an initial 10 min at 94°C, followed by 40 cycles at 94°C for 30 sec, 30 sec at 58°C and 30 sec at 72°C. The reaction was run in a 96 well microtitre plate (Bio-Rad) in an iQ 5 thermal cycler (Bio-Rad). Samples were run in triplicate.

### 2.4 Standard curve analysis

A standard curve was obtained by linear regression analysis between threshold cycle (Ct) over the amount of the DNA of each of the three replicates of the standard dilutions (Supplementary Figure 1). Data analysis and interpretation were done automatically by the software. PCR efficiency was calculated by the formula:


E=e-ln/10-s1

A PCR efficiency of 100 ± 5% using the standard curve constructed with serial dilutions of genomic DNA is sufficient for further quantification.

### 2.5 Environmental data

Environmental data (temperature, humidity, and rainfall) were obtained from the Pakistan meteorological department^1^ and from online resources.^2^

## 3 Results

### 3.1 Measurement of environmental variables

**FIGURE 1:**
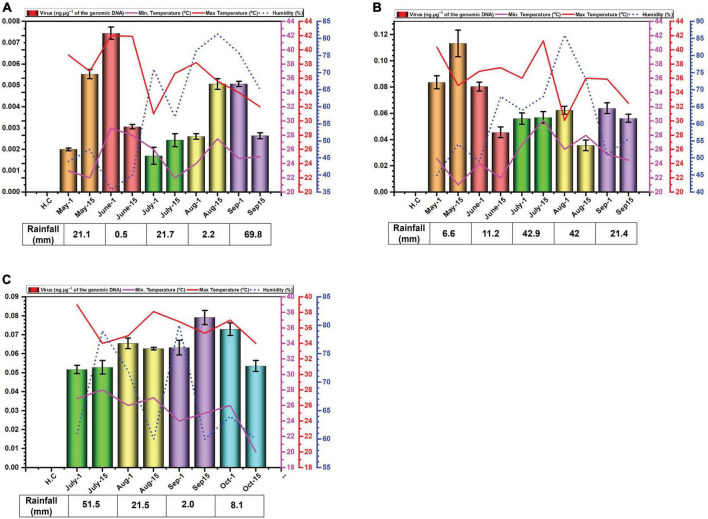
Environmental cues (temperature, average humidity, and average rainfall) observed across the three cotton growing periods and qPCR analysis of the levels of virus in *B. tabaci* insects collected from cotton in Faisalabad over the cotton growing periods 2014 **(A)**, 2015 **(B)** and 2016 **(C)**. qPCR data are presented as mean ± standard deviation.

The data for environmental cues (temperature, relative humidity, and rainfall) at the sampling location exhibited variations across the study period. In 2014, average monthly maximum temperatures ranged from 34.6°C in September to 41.9°C in June (Figure 1A). While in 2015, with average highs reaching 40.4°C in May low 35.9 in °C in September (Figure 1B). In 2016, maximum recorded temperature was 37.2°C in July and minimum was 34.7°C in October (Figure 1C). Relative humidity displayed considerable variation throughout the experiment. In 2014, it peaked in July and September, exceeding 60%. In 2015 and 2016, it was highest in July and August with exceeding 60% relative humidity (Figure 1C).

Rainfall patterns differed significantly across the three years. In 2014, a substantial rainfall event occurred in September (69.8 mm) (Figure 1A). In 2015 and 2016, the highest rainfall was observed in July and was 42.9 and 51.5 mm, respectively (Figures 1B, C).

### 3.2 Quantification of begomovirus DNA

**FIGURE 2:**
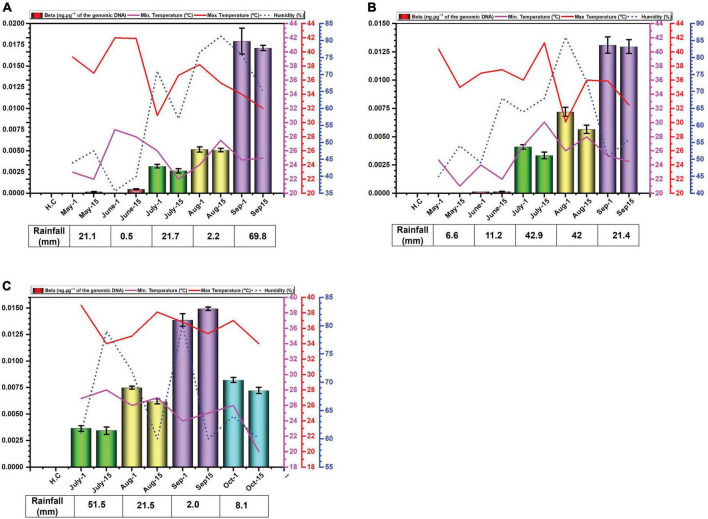
Environmental cues (temperature, average humidity, and average rainfall) observed across the three cotton growing periods and qPCR analysis of the levels of betasatellite in *B. tabaci* insects collected from cotton in Faisalabad over the cotton growing periods 2014 **(A)**, 2015 **(B)** and 2016 **(C)**.

Whitefly samples were collected each month starting from May to September in the year 2014, 2015 and from July to October in the year 2016. The late sowing in 2016 was attributed to a lack of water for irrigation. The results of the qPCR determination of the amounts of virus DNA in the samples collected are given in Figure 2. The results for the amounts of viral DNA in insects firstly showed considerable variation between the two samples collected at each time point, suggesting a great variation in the amounts of virus carried by each insect. Although in the first year of sampling (2014) the amounts of virus in insects possibly showed a peak in June and a slight trough in July this was far from convincing. In 2014, viral titres exhibited similar levels in the second sampling of July and the first sampling of August, as well as the second sampling of August and the first of September (Figure 1A). In 2015, the amounts of virus in insects potentially peaked in May, followed by a period of comparable levels from the second sampling of June to the first sampling of August (Figure 1B). In contrast to the previous years, viral titres in 2016 appeared to peak in September. Notably, both samples of July, the second of August, and the first of September all displayed comparable viral DNA titres (Figure 1C). Overall, there was little variation in the amounts of viral DNA in insects over the sampling periods. Striking are the very low amounts of viral DNA detected in insects in the year 2014 in comparison to the two following year—a factor of 10-fold difference.

### 3.3 Quantification of betasatellite DNA

The amounts of betasatellite DNA detected in insects exhibited a distinct pattern of accumulation throughout the study period. Initially, the amounts detected were very low, followed by a gradual built-up during the sampling period. In 2014, betasatellite DNA peaked in September. A gradual increase throughout the season lacked significant variation between individual monthly sampling (Figure 2A). In 2015, a similar pattern emerged, with a key difference: betasatellite titres declined during the second sampling of each month (Figure 2B), suggesting a potential biphasic cycle within each month. Noticeably year 2016, with delayed planting schedule, displayed a unique trend. The amount of betasatellite DNA detected at the first sampling (July) was significantly higher than in the previous two years, potentially reflecting the impact of planting time on vector-virus dynamics. However, the levels and also dropped off at the last sampling in October (Figure 2C). Also, in contrast to the figure for virus DNA, the amount of variation between the two samples at each sampling showed less variation than was the case for viral DNA.

## 4 Discussion

Geminiviruses are transmitted by their insect vector in a persistent, circulative manner. Viruses of the genus *Begomovirus* are transmitted exclusively by the whitefly, *B. tabaci*. Although for the vast majority of begomoviruses the association with *B. tabaci* is non-propagative, the virus does not undergo replication in the whitefly, for Tomato yellow leaf curl virus there is some evidence that, in fact, the virus may undergo replication in (at least some) biotypes (cryptic species) of *B. tabaci* (Pakkianathan et al., 2015; Wang et al., 2016). The circulative pathway of begomoviruses in *B. tabaci* has been extensively studied (Rosell et al., 1999; Ghanim et al., 2001a; Czosnek et al., 2002). Whiteflies are phloem feeders and *B. tabaci* ingests begomoviruses through its stylets whilst feeding on the phloem of infected plants. Virions pass through the food canal, the esophagus, the filter chamber and into the mid-gut. In the filter chamber and/or anterior mid-gut the virus crosses the gut wall into the haemolymph, which circulates throughout the insect. Carried in the haemolymph the virus translocates into the primary salivary glands and is egested with the saliva along the salivary canal into the plant phloem. The time taken from ingestion to ultimate egestion in the saliva is known as the “latent period” and is a time during which the insect is unable to infect—transmit the virus to–healthy plants upon which it feeds (Czosnek et al., 2017).

Detection of begomoviruses and their path in insect vector whiteflies has been studied extensively (Ghanim et al., 2001b; Ghanim, 2014; Czosnek et al., 2017). Molecular hybridization methods have been used to detect pathogens, but the PCR-based methods are more sensitive and allow quantification as reviewed by Accotto and Noris (2007). qPCR has an advantage over conventional PCR due to its greater sensitivity (Mumford et al., 2000; Korimbocus et al., 2002; Boonham et al., 2004) and for this reason has become the method of choice to detect viruses in insect vectors (Boonham et al., 2002; Fabre et al., 2003; Noris and Miozzi, 2015). qPCR analysis confirms whiteflies’ remarkable efficiency as virus carriers. They acquire millions and transmit billions of begomovirus particles within minutes (Roy et al., 2021). Notably, whiteflies feeding on infected zucchini harbored significantly more ToLCNDV-ES and transmitted it with much greater efficiency (96%) compared to those feeding on tomatoes (2%) (Janssen et al., 2022).

A general assumption, for the infection of crops by begomoviruses, is that *B. tabaci* “overwintering” on weeds/ornamentals or winter crops migrate into the new plantings and bring the virus with them to establish infection. The field in Faisalabad where this study was conducted is in an area where predominantly chickpea, lentil, and wheat are grown over the winter months. These are not hosts of the virus complex that causes CLCuD. They are instead hosts to other geminiviruses, such as Chickpea chlorotic dwarf virus for chickpea and lentil (Nahid et al., 2008; Kraberger et al., 2015) or, in the case of wheat, have not so far been shown to be a host of geminiviruses in this area. It would seem not unreasonable to assume that, at the time of planting, the majority of virus inoculum carried by *B. tabaci* insects originates from plants which are not cotton, and are not good hosts for the viruses causing CLCuD (such as chickpea and lentil). However, there are exceptions to this, such as *Hibiscus rosa-sinensis*, which is grown as an ornamental in gardens and on roadside verges. *H. rosa-sinensis*, a plant of the family *Malvaceae*, is a host of the virus complex causing CLCuD (Sattar et al., 2013; Akhtar et al., 2014). Also, in the area where the insect samples were collected are several small plots of ratoon cotton; cotton plants from the previous year maintained for seed multiplication and invariably showing symptoms of CLCuD. The sole presence of Asia II-1 whiteflies, known to transmit only the CLCuKoV-Bur strain (Masood and Briddon, 2018; Pan et al., 2018), suggests minimal impact from other unidentified CLCuD strains in this region.

We excluded alphasatellite based on prior findings (Iqbal et al., 2023) where its titre showed a non-significant negative correlation with seed cotton yield (*p* ≤ 0.05), while betasatellite titre showed a significant and strong negative correlation. Furthermore, betasatellite titre was identified as an important variable affecting plant performance, unlike the virus and alphasatellite titre. The basis for the study was the hypothesis that the virus and betasatellite titre in the vector *B. tabaci* would change over the growing season of cotton. The results obtained showed that this is the case for betasatellite DNA levels but are somewhat ambiguous for the virus DNA levels changing over the period of the study for each year. Particularly for 2016 but also to some degree for 2015, the levels of virus detected in insects were not significantly different as evidenced by largely overlapping error bars for the monthly values. Noticeable also is the variation between samples collected in the same month, suggesting that there is significant variation in the amount of virus carried by individual insects at each time period. Nevertheless, based on the results from 2014, there appears to be a peak in virus DNA levels for June, a trough in July followed by another peak in August/September. This would suggest that insects migrating into cotton early in the season (May) carry low amounts of virus, suggesting that the plants upon which the insects fed prior to migrating into cotton contained low amounts of virus and betasatellite.

Very low virus amounts were acquired by insects in 2014, relative to the other two years. The possible reasons for this are unclear. However, 2015 was a particularly bad year for cotton production in Pakistan with record losses (production estimated at 526 kg.ha^–1^, a drop of 32.74% over the previous year).^3^ Similarly, the production in 2016 was lower (671 kg.ha^–1^) than in 2014 (a record year with production estimated at 782 kg.ha^–1^) but nevertheless better than in 2015. Possibly, there was a particularly virulent CLCuD complex after 2014, although there is no evidence to suggest that this was the case.

What is quite noticeable is that the virus DNA levels are not mirrored by the betasatellite DNA levels. Possibly this is not surprising since there is no evidence to suggest that *in planta* betasatellite levels are tied to virus levels despite the fact that betasatellite replication depends upon virus-encoded Rep (Saunders et al., 2000; Briddon et al., 2001). This lack of correlation between virus and betasatellite levels in plants was evident in the recent study of the correlation between virus/betasatellite levels in plants in relation to the severity of CLCuD symptoms (Shafiq et al., 2017; Iqbal et al., 2023). This contrasts with the other family of plant-infecting ssDNA viruses, the multipartite genome nanoviruses, where the relative levels of each component seems strictly controlled in both plants and insect vector (Sicard et al., 2013).

In contrast the virus DNA levels, which seem to rise and fall during the season (possible due to ambient temperatures), the DNA levels of the betasatellite appear to increase gradually during the growing season, peaking at the end of the sampling (harvest time). Only for the 2016 season, the year that planting was delayed, was there a drop-off in betasatellite level at the last sampling time. This possibly might occur due to senescence and/or a reduction in plant growth this late in the year with low temperatures and shortening day-length. Whitefly adults typically favor young leaves, likely due to lower plant defenses and higher nitrogen content (Ahmed et al., 2007; El-Zahi et al., 2012). Interestingly, *B. tabaci* on cotton are less likely to stay on and lay eggs (oviposit) on leaves with high light intensity (L*) and a yellow color (do Prado et al., 2016). However, the difference in acquired virus and betasatellite DNA levels is difficult to explain. Betasatellite DNA is encapsidated in virus-encoded coat protein (CP), allowing it to be acquired and transmitted by the vector (Tabein et al., 2013). Since both viral and betasatellite particles solely consist of CP and their respective DNA, it is difficult to see why betasatellite accumulate to higher levels within insects. Insect vectors of circulatively transmitted plant viruses can be seen as efficient sieves which selectively “filter-out” nutrients and virus particles from a large volume of plant (phloem) sap. Virus particles are selectively taken up in the filter chamber and/or anterior mid-gut by endocytosis and transported into the haemolymph. The interaction at the gut wall is selective, only begomoviruses (in the case of *B. tabaci*) being able to interact with specific receptors to pass into the haemocoel—most likely by clathrin-mediated endocytosis (Pan et al., 2017). The specificity is determined, on the virus side, by amino acid sequences on the CP (Briddon et al., 1990; Noris et al., 1998; Höhnle et al., 2001; Caciagli et al., 2009). The receptor(s) within the insect responsible for specific begomovirus up-take remain unclear, although several proteins have been implicated in the process (Czosnek et al., 2017). The same virus CP amino acid sequences and insect receptors are also believed to be involved in the specific transport of the virus from the haemocoel into the salivary secretions (Höfer et al., 1997; Noris et al., 1998; Höhnle et al., 2001; Caciagli et al., 2009; Wei et al., 2014). These factors would thus suggest that the relative amounts of virus and betasatellite containing particles acquired by the insect should mirror the amounts present in the plants on which the insects feed. Although the relative amounts of virus and betasatellite DNA in infected plants has been examined at single time points, such as in the study of CLCuD affected cotton (Shafiq et al., 2017), no studies have so far examined the relative amounts virus and betasatellite DNA across a growing season. The relationship between virus and betasatellite DNA in infected plants relative to the virus and betasatellite DNA in insects feeding on the plants thus remains unclear. The results might thus suggest that particles containing betasatellite DNA are preferentially acquired, relative to particles containing viral DNA, or, alternatively, are less readily excreted by the insect. A possible explanation for this could be that betasatellite DNA, being half the size of the helper begomovirus genome, is encapsidated in isometric rather than geminate particles. For Maize streak virus, isometric particles have been shown to encapsidate approx. half genome length subgenomic (also known as “defective”) DNAs (Casado et al., 2004). Betasatellite DNA could thus be encapsidated in isometric particles which might be treated differently by the acquisition/transmission system of the insect than geminate particles. For example, there might be more retention sites in the insect (on GroEL) for isometric particles than geminate particles. This apparent anomaly requires further investigation.

With limited numbers of receptors in the insect to acquire begomoviruses (the gut-haemocoel boundary) and limited numbers of receptors to excrete begomoviruses (the haemocoel-salivary gland boundary) *B. tabaci* insects appear to have a maximum capacity for virus; the implication being that *B. tabaci* has a mechanism to control the amounts of virus in the insect (Zeidan and Czosnek, 1991; Caciagli and Bosco, 1997; Becker et al., 2015; Ning et al., 2015; Czosnek et al., 2017). The results obtained here with the CLCuD complex suggest that at no point during the cotton growing season, or at the very least for the majority of the season, is this maximum reached. This might indicate that the plants upon which the insects are feeding do not contain enough virus/betasatellite to saturate the insect, or the environmental conditions are sub-optimal for the acquisition of virus by the insect. No studies have so far examined the effects of, in particular, the temperature on the acquisition and transmission of begomoviruses by *B. tabaci*. Such a study would be difficult to conduct since the temperature has a significant effect on plant growth which would also have an effect on available virus/betasatellite for acquisition. However, this problem could be overcome by feeding insect through membranes on purified virus; assuming that begomovirus stability is not adversely affected by temperature. Membrane feeding insects has previously been used to show that exchange of the coat protein of a begomovirus for that of a curtovirus changes vector specificity from *B. tabaci* to the leafhopper vector of the curtovirus (Briddon et al., 1990).

Environmental variables have a significant effect on plant growth (Ullah et al., 2016) and on CLCuD severity and incidence (Khan and Khan, 2000; Khan et al., 2015; Maharshi et al., 2017). Similarly, environmental conditions have a great effect on *B. tabaci*, affecting reproduction and thereby population numbers (Khan et al., 2015). However, what effects the environment has on the levels of begomoviruses acquired by *B. tabaci* has not been investigated. This issue requires investigation.

Overall, the study described here has raised more questions than it has answered. The amounts of at least the betasatellite appear to increase gradually during the season whereas for the amount of virus the situation is less clear, the 2014 analysis appearing to suggest that there are two peaks of virus titre. Nevertheless, the results would seem to support the idea that controlling insects early in the season, by for example treatments with insecticides would be more effective than treatments later in the season, to prevent the build-up of inoculum in the insect. Later in the season, most plants are symptomatically infected and insects appear to contain large amounts of inoculum. Clearly, the study conducted here needs repeating to, particularly, establish what is happening with respect to virus levels in whiteflies and try to establish the effects of environmental variables on the levels of virus/betasatellite in whiteflies. It would also be interesting to determine the levels of alphasatellite, the third part of the CLCuD complex, in whiteflies during the cotton growing season.

## Data availability statement

The original contributions presented in this study are included in this article/Supplementary material, further inquiries can be directed to the corresponding author.

## Ethics statement

The manuscript presents research on animals that do not require ethical approval for their study.

## Author contributions

ZI: Funding acquisition, Investigation, Software, Writing – original draft, Writing – review & editing. MM: Formal analysis, Investigation, Methodology, Writing – original draft, Writing – review & editing. MS: Investigation, Software, Writing – review & editing. RB: Conceptualization, Project administration, Supervision, Writing – original draft, Writing – review & editing.

## Footnotes

1
http://namc.pmd.gov.pk/

2
www.timeanddate.com

3
https://www.indexmundi.com/agriculture/?country=pk&commodity=cotton&graph=yield
