# Supplementary material for: Temporal changes in the levels of virus and betasatellite DNA in B. tabaci feeding on CLCuD affected cotton during the growing season
Source: Front Microbiol. 2024 May 22;15:1410568. doi: 10.3389/fmicb.2024.1410568 (PMC11150673; doi:10.3389/fmicb.2024.1410568)
Supplement: Supplementary file 1 [file Presentation_1.pdf]

Supplementary Material

Temporal changes in the levels of virus and betasatellite DNA in *B. tabaci* feeding on CLCuD affected cotton during the growing season

Zafar Iqbal[1*], Mariyam Masood[2*], Muhammad Shafiq[3], Rob W Briddon[4]

[1] Central Laboratories, King Faisal University, Al-Ahsa 31982, Saudi Arabia. zafar@kfu.edu.sa

[2] Department of Zoology, Government College Women University, Madina town, Faisalabad, Pakistan. joinmariyam@hotmail.com

[3] Department of Biotechnology, University of Management and Technology, Sialkot Campus, Sialkot P.O. Box 51340, Pakistan. shafiq.4721@gmail.com

[4] Agricultural Biotechnology Division, National Institute for Biotechnology and Genetic Engineering, Faisalabad, Pakistan. rob.briddon@gmail.com

* Correspondence and equal contribution

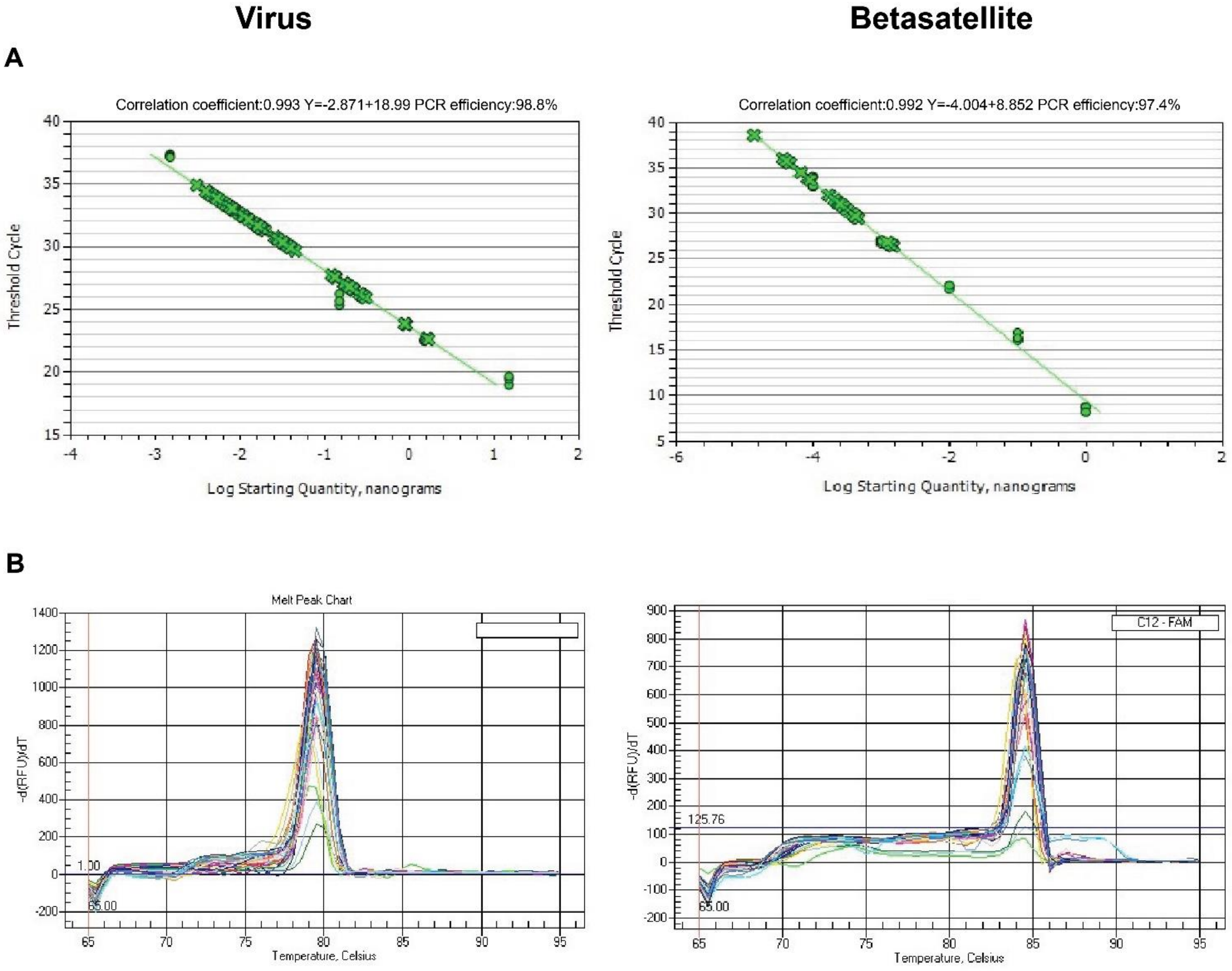

Figure S1. Standard curve (A) for amplification of Begomovirus (left) and betasatellite (right). Melt peak chart (B) and melt curve chart for estimation of the titre of begomovirus (right) and betasatellites (left). The standard curves plot log of starting DNA quantity against threshold cycle [C(T)] cycle. The melt curves plots negative rate of change of fluorescence [-d(RFU)/dT] against temperature.
